# Lipid raft involvement in yeast cell growth and death

**DOI:** 10.3389/fonc.2012.00140

**Published:** 2012-10-10

**Authors:** Faustino Mollinedo

**Affiliations:** Instituto de Biología Molecular y Celular del Cáncer, Centro de Investigación del Cáncer, Consejo Superior de Investigaciones Científicas - Universidad de SalamancaSalamanca, Spain

**Keywords:** lipid rafts, membrane domains, ergosterol, yeast, *S. cerevisiae*, ion homeostasis, nutrient transporters, cell death

## Abstract

The notion that cellular membranes contain distinct microdomains, acting as scaffolds for signal transduction processes, has gained considerable momentum. In particular, a class of such domains that is rich in sphingolipids and cholesterol, termed as lipid rafts, is thought to compartmentalize the plasma membrane, and to have important roles in survival and cell death signaling in mammalian cells. Likewise, yeast lipid rafts are membrane domains enriched in sphingolipids and ergosterol, the yeast counterpart of mammalian cholesterol. Sterol-rich membrane domains have been identified in several fungal species, including the budding yeast *Saccharomyces cerevisiae*, the fission yeast *Schizosaccharomyces pombe* as well as the pathogens *Candida albicans* and *Cryptococcus neoformans*. Yeast rafts have been mainly involved in membrane trafficking, but increasing evidence implicates rafts in a wide range of additional cellular processes. Yeast lipid rafts house biologically important proteins involved in the proper function of yeast, such as proteins that control Na^+^, K^+^, and pH homeostasis, which influence many cellular processes, including cell growth and death. Membrane raft constituents affect drug susceptibility, and drugs interacting with sterols alter raft composition and membrane integrity, leading to yeast cell death. Because of the genetic tractability of yeast, analysis of yeast rafts could be an excellent model to approach unanswered questions of mammalian raft biology, and to understand the role of lipid rafts in the regulation of cell death and survival in human cells. A better insight in raft biology might lead to envisage new raft-mediated approaches to the treatment of human diseases where regulation of cell death and survival is critical, such as cancer and neurodegenerative diseases.

## INTRODUCTION

Apoptosis is an intrinsic cell death process that plays critical roles in the normal development and health of multicellular organisms. However, in the last years, growing evidence suggests that apoptosis-like cell death also occurs in a number of unicellular organisms, including yeast (Madeo et al., 2002, 2004; Wissing et al., 2004; Pereira et al., 2008; Carmona-Gutierrez et al., 2010; Sousa et al., 2011). Some features of apoptotic-like cell death can be induced in yeast following stress conditions, such as acetic acid (Ludovico et al., 2001, 2002), membrane-permeable C2-ceramide (Carmona-Gutierrez et al., 2011), hydrogen peroxide (H_2_O_2_; Madeo et al., 1999; Ribeiro et al., 2006), hyperosmotic (Silva et al., 2005; Ribeiro et al., 2006), and NaCl (Huh et al., 2002; Wadskog et al., 2004) stress.

Cell membranes are structurally heterogeneous, composed of discrete domains with unique physical and biological properties. The concept of the organization of membrane lipid components into domains (Karnovsky et al., 1982), and the subsequent demonstration of the existence in the plasma membrane of a particular type of microdomain enriched in sterols and sphingolipids, named as lipid rafts (Simons and Ikonen, 1997; Brown and London, 2000; Simons and Toomre, 2000; Ikonen, 2001; Maxfield, 2002), have profoundly changed our view of membrane organization and membrane-regulated processes. Membrane domains can form through a number of mechanisms involving lipid–lipid and protein–lipid interactions.

Despite lipid rafts have different sizes depending on the membrane composition, a consensus definition of a lipid raft developed at the 2006 Keystone Symposium of Lipid Rafts and Cell Function, held in Steamboat Springs (CO, USA), concluded that “membrane rafts are small (10–200 nm), heterogenous, highly dynamic, sterol- and sphingolipid-enriched domains that compartmentalize cellular processes. Small rafts can sometimes be stabilized to form larger platforms through protein–protein and protein–lipid interactions” (Pike, 2006). In resting cells, rafts appear small and unstable, and the current consensus is that their sizes are smaller than the optical diffraction limit (250 nm). Following stimulation, raft-preferring proteins are clustered, inducing larger and stabilized rafts, likely through lipid lateral diffusion and coalescence of small raft units.

A critical issue in the studies of raft biology lies in the difficulty to visualize rafts in living cells, unless they coalesce in large raft platforms. Thus, most of the evidence for their identification relies on indirect methods, such as the use of non-ionic detergent extraction. On these grounds, rafts are usually defined as the insoluble fraction or detergent-resistant membrane (a.k.a., DRM) after non-ionic detergent solubilization at 4^°^C, which can be isolated by flotation in density gradients. The association of a protein with cholesterol-rich rafts is strengthened when it becomes detergent-soluble after depletion of cholesterol from the membrane by the use of methyl-β-cyclodextrin or other agents. Nevertheless, the physiological existence of rafts has been challenged by a number of criticisms (Munro, 2003; Lichtenberg et al., 2005; Lingwood and Simons, 2007; Simons and Gerl, 2010), in particular regarding the use of detergents that could lead to artifacts and misinterpretations since, for instance, Triton has been shown to promote the formation of ordered domains in model bilayers (Heerklotz, 2002). Also, a main concern has been raised on the diverse effects that might be expected by depleting cholesterol from the membrane because cholesterol has important functions in the whole plasma membrane, apart from forming lipid rafts. On these grounds, the functional involvement of rafts as well as the raft localization of proteins based only on the use of detergents and cholesterol depletion has been challenged, and therefore caution should be taken before assigning a role of rafts in different biological processes. In addition, the evidence for the presence of rafts in the plasma membrane of living cells has, until recently, not been compelling, thus raising some doubts about the physiological existence of rafts. However, the advent of new microscopy techniques has finally demonstrated the existence of rafts in the cell. The use of stimulated emission depletion (STED) microscopy has proved that sphingolipids and glycosylphosphatidylinositol (GPI)-anchored proteins are transiently trapped in cholesterol-dependent molecular complexes in live cells (Eggeling et al., 2009). In this regard, the application of novel technologies, such as fluorescence resonance energy transfer (FRET), fluorescence polarization anisotropy (FPA), total internal reflection fluorescence (TIRF) microscopy, single quantum dot tracking, single particle tracking (SPT), and fluorescence correlation spectroscopy (FCS), have provided evidence for the localization of GPI-anchored proteins and other lipid-modified proteins in cholesterol-dependent clusters (Kusumi et al., 2004; Sharma et al., 2004; Lenne et al., 2006; Marguet et al., 2006; Vyas et al., 2008; Pinaud et al., 2009). In addition, near-field scanning optical microscopy (NSOM) in combination with quantum dots showed that T cell stimulation triggers the organization of T cell receptors in nanodomains in live cells (Zhong et al., 2009; van Zanten et al., 2010). The development of the above high temporal and spatial resolution techniques has allowed to locate different molecular constituents in membrane domains with reduced mobility. The combined use of different biophysical, biochemical, and genetic technologies are now providing evidence demonstrating the existence of sterol-dependent membrane raft domains as well as their role in critical physiological functions. Despite some technical and conceptual limitations, as stated above, resistance to non-ionic detergent solubilization, together with flotation in gradient density centrifugation and manipulation of sterol, remain as the most widely used techniques for studying lipid rafts.

There are a wide number of reports showing that rafts in mammalian cells house proteins involved in cell survival and growth, as well as in the proper functioning of immune system receptors (Simons and Toomre, 2000; Szoor et al., 2010). In this regard, cancer cells, usually showing an increased ability to proliferate and survive, have been found to have higher levels of cholesterol (Dessi et al., 1994; Kolanjiappan et al., 2003; Freeman and Solomon, 2004; Tosi and Tugnoli, 2005) and cholesterol-rich lipid rafts (Li et al., 2006) than their normal counterparts. Nevertheless, in the last decade, a number of death receptors and downstream apoptotic signaling molecules have also been localized in cholesterol- and sphingolipid-rich lipid rafts in cancer cells (Gajate and Mollinedo, 2001, 2005, 2007; Gajate et al., 2004; Mollinedo and Gajate, 2006; Reis-Sobreiro et al., 2009; Mollinedo et al., 2010a). The localization of the death-inducing signaling complex (DISC), a major apoptotic complex containing Fas/CD95 death receptor, Fas-associated death domain-containing protein (FADD) and procaspase-8, in lipid rafts has been shown by electron microscopy in T cell leukemic cells, when they are engaged to undergo apoptosis (Gajate et al., 2009a). In addition, raft nanodomains have been shown, following a FCS strategy, to be present in both the outer and inner leaflets of the plasma membrane and to play a crucial role in triggering the survival phosphatidylinositol-3 kinase/Akt signaling pathway, by facilitating Akt recruitment and activation upon phosphatidylinositol-3,4,5-triphosphate accumulation in the plasma membrane (Lasserre et al., 2008). On these grounds, mammalian cell lipid rafts behave as platforms that can house different, and even opposite signaling processes, such as survival and apoptosis, and therefore these membrane domains play a critical role in the modulation of cell signaling that regulates cell fate.

Lipid rafts have also been identified in yeast as membrane domains enriched in ergosterol and sphingolipids (Wachtler and Balasubramanian, 2006). The budding yeast *Saccharomyces cerevisiae* is one of the best characterized eukaryotic organisms. In spite of its simplicity as a free-living unicellular fungus, yeast cells are similar to higher eukaryotes regarding the cell structure and several physiological processes. Due to its genetic tractability and increasing wealth of accessible data, yeast has become a model system of choice for the study of different physiological processes occurring in mammalian cells. In this regard, yeast could be an interesting biological system to analyze the role of lipid rafts in both survival and cell death responses, despite yeast lack death receptors and most of the typical apoptotic signaling molecules present in mammalian cells.

## RAFTS IN YEASTS

Regarding the lipid constituents of lipid rafts, and although the lipid levels vary between different cell types, the plasma membrane of the mammalian cell usually contains, on a molar basis, about 30–40% cholesterol and 10–20% sphingomyelin of plasma membrane lipids, while glycosphingolipids are usually present at low levels (Lange et al., 1989; van Meer, 1989). However, yeasts do not have sphingomyelin, but instead have inositol phosphosphingolipids, which may function as orthologs of mammalian sphingomyelin (Matmati and Hannun, 2008). In addition, unlike mammalian cells that have cholesterol, yeast contain ergosterol, serving the same function as cholesterol in animal cells. Ergosterol is even a better raft former than cholesterol (Xu et al., 2001). Studies on the generation of model membranes from yeast total lipid extracts led to the conclusion that formation of membrane domains depended on specific interactions between yeast sphingolipids and ergosterol (Klose et al., 2010). This selective interaction between yeast sphingolipids and ergosterol results in phase separation into membrane domains with liquid ordered- and liquid disordered-like properties, that is in raft formation (Klose et al., 2010). Thus, whereas lipid rafts in mammalian cells contain cholesterol and sphingomyelin or glycosphingolipids (Simons and Ikonen, 1997), raft domains in *S. cerevisiae* contain ergosterol and complex sphingolipids (Kubler et al., 1996; Bagnat et al., 2000), including inositol-phosphoceramide (IPC), mannose-inositol-phosphoceramide (MIPC), and mannose-(inositol phosphate)_2_-ceramide (M(IP)2C) (Dickson et al., 2006).

Lipid rafts are enriched in sterols, composed of a four-ring structure with an aliphatic tail that can pack tightly with the lipid acyl chains of sphingolipids to create a compacted region of condensed bilayer termed the liquid-ordered state (Munro, 2003; Megha et al., 2006). Because of its rather unique lipid composition, lipid rafts are more resistant to extraction with cold non-ionic detergents, and therefore they were originally defined as DRMs, due to their relative insolubility in cold non-ionic detergents (London and Brown, 2000; Maxfield, 2002; Simons and Gerl, 2010). In the yeast, rafts have also been defined biochemically as DRMs and proven to be critical for protein sorting through the endoplasmic reticulum and Golgi apparatus (Bagnat et al., 2000, 2001; Bagnat and Simons, 2002). The terms lipid raft, liquid-ordered domain, and DRM are widely used indistinctly and are suggested to refer to the same chemico-biological entity. However, in a very strict way, they might have different implications and caution should be taken (Lichtenberg et al., 2005).

The yeast sphingolipid is peculiar in that it contains a saturated very long-chain fatty acid with 26 carbon atoms (Schneiter et al., 1999, 2004), which is synthesized and coupled to raft-located proteins, like the proton pumping ATPase Pma1p, already in the endoplasmic reticulum. Then, the resulting protein–lipid complex is transported and sorted as an entity to the plasma membrane. This rather long C26 fatty acid is required for proper assembly of the protein–lipid complex and transport to the membrane, as a shortening to C22 fatty acid by mutations impairs raft association of Pma1p, and induces Pma1p degradation by rerouting the ATPase enzyme from the plasma membrane to the vacuole (Gaigg et al., 2006; Toulmay and Schneiter, 2007).

Another important difference between mammalian cells and yeast cells lies in that lipid raft formation occurs primarily in the Golgi apparatus in mammals (Brown and Rose, 1992), whereas it takes place in the endoplasmic reticulum in yeast, where is suggested that proteins associate with yeast rafts (Bagnat et al., 2000). In both yeast and mammalian cells, sphingolipids and sterols are mainly present in the plasma membrane (Lange et al., 1989; Patton and Lester, 1991; Zinser and Daum, 1995), but these molecules are synthesized in compartments of the early secretory pathway (Daum et al., 1998; Futerman and Riezman, 2005), and therefore raft-located proteins could be recruited into these domains in other subcellular structures distinct from the plasma membrane. Thus, in yeast newly synthesized Gas1p, a GPI-anchored protein, and Pma1p have been found to be recruited to lipid rafts in the endoplasmic reticulum (Bagnat et al., 2000; Lee et al., 2002).

Because a major constituent of lipid rafts is sterol, the naturally fluorescent sterol-binding antibiotic filipin has been widely used to detect regions with high sterol content in the plasma membrane of fungi. The use of this compound has led to the identification of large sterol-rich domains (SRDs) in the plasma membranes of fungi (Wachtler et al., 2003; Alvarez et al., 2007), ranging from about 3 to 15 μm^2^. Due to the dynamic nature of lipid rafts and to their ability to aggregate, clusters of rafts in mammalian cells can be formed under different stimuli to lead to raft platforms as big as the ones reported for some fungi (Ausili et al., 2008). In this regard, it might be envisaged that SRDs in yeasts correspond to clusters of sterol-rich rafts or raft platforms, which are required for the accomplishment of specific functions. This clustering of rafts may be critical for the proper onset of certain cell functions, which might require the concentration of a great amount of proteins in specific sites of the cell, thus leading to cell movement, cytokinesis, etc. On these grounds, lipid rafts act as dynamic and mobile platforms that transport the required proteins at the proper place to act.

Sterol-rich membrane domains have been identified in several fungal species, including the budding yeast *S. cerevisiae* (Kubler et al., 1996; Bagnat et al., 2001), the fission yeast *Schizosaccharomyces pombe* (Wachtler et al., 2003), as well as the pathogen yeasts *Candida albicans* (Martin and Konopka, 2004; Alvarez et al., 2007) and *Cryptococcus neoformans* (Siafakas et al., 2006; Alvarez et al., 2007), having being involved in a number of important processes like mating (Nichols et al., 2004; Proszynski et al., 2006), cytokinesis (Rajagopalan et al., 2003; Wachtler et al., 2003), and hyphal formation (Martin and Konopka, 2004).

Sterol-rich domains are polarized in the rod-shaped *S. pombe* throughout the vegetative life cycle in a cell cycle-dependent way, namely they are located to the growing cell ends during interphase, and to the medial zone where cells undergo cytokinesis, as well as at the tips of mating projections (Rajagopalan et al., 2003; Wachtler et al., 2003; Wachtler and Balasubramanian, 2006; Alvarez et al., 2007). Thus, in fission yeast, rafts localize to regions of polarized growth and to the division site. Unlike to what happens in *S. pombe*, sterols are distributed uniformly throughout the plasma membrane in the vegetative life cycle of the budding yeast *S. cerevisiae*. However, filipin-stained rafts are also detected at the tips of cells induced with mating pheromone (Bagnat and Simons, 2002; Proszynski et al., 2006; Alvarez et al., 2007). In the human pathogens *C. neoformans* and *C. albicans*, sterols are concentrated at the leading edges of mating projections, at the actively growing sites at bud tips, at sites of septation, and at the tip of hyphal growth (Martin and Konopka, 2004; Alvarez et al., 2007).

## RAFTS IN YEAST PATHOGENS

Proteomic analysis in DRMs from *C. albicans* led to the identification of 29 proteins, including the well-known lipid raft marker in *S. cerevisiae* Pma1p (Insenser et al., 2006). Surprisingly, only three proteins (~10%) were typically located in the plasma membrane, whereas most of raft-located proteins were usually present in internal membranes, including proteins located in mitochondrial (31%), Golgi (7%), and endoplasmic reticulum (7%) membranes. This could support the existence of raft domains in the membranes of mitochondria, endoplasmic reticulum, and Golgi, as reported by different researchers (Bini et al., 2003; Mielenz et al., 2005; Mollinedo et al., 2011). The proteins located in *C. albicans* rafts were involved in a number of biological processes, including lipid metabolism, cell wall biogenesis, protein metabolism, electron transport, and ATP synthesis (Insenser et al., 2006). In addition heat shock proteins were also present (Insenser et al., 2006), similarly to what has been observed in mammalian cells (Nieto-Miguel et al., 2008). Likewise, ATP synthase was also located at the raft membranes in *C. albicans* (Insenser et al., 2006), and a similar raft location for this enzyme has been found in proteomic studies conducted in mammalian cells (Bae et al., 2004), leading to the suggestion that this protein might be located in plasma membrane rafts as well as in mitochondria (Bae et al., 2004). Furthermore, it is worth mentioning the presence in *C. albicans* lipid rafts of a series of proteins involved in lipid metabolism and multidrug efflux, such as: Erg11p and Scs7p, involved in the lipid metabolism of major raft components (ergosterol and ceramide; Insenser et al., 2006); Rta2p, a translocase that moves sphingolipid long chain bases from the inside to the outside membrane (Wang et al., 2012); and the ATP-binding cassette (ABC) multidrug transporter CaCdr1p (Pasrija et al., 2008). The presence of cytosolic proteins in the *C. albicans* rafts suggest that protein–protein interactions could play a major role in bringing soluble proteins to the raft domains.

*Candida albicans*-associated bloodstream infections are linked to the ability of this yeast to form biofilms (Mukherjee et al., 2005). These latter are aggregates of microbial cells, adhering to each other, which are embedded within a self-produced matrix of extracellular polymeric substances, usually made up of extracellular DNA, proteins, and polysaccharides, and represent a common mode of microbial growth. Microbes growing as biofilm are highly resistant to commonly used antimicrobial drugs. *Candida* biofilms associated with indwelling devices provide a protected niche for the fungal cells, where they can evade the host immune system, and are especially problematic due to their inherent resistance to commonly used antifungal agents (Chandra et al., 2007). The microbial cells growing in a biofilm are physiologically distinct from planktonic cells of the same organism, which are single-cells floating in a liquid medium. Biofilm formation by *Candida* species is believed to contribute to invasiveness of these fungal species, and there is a correlation between *C. albicans* biofilms and fungal pathogenesis. By using lipidomics, a significant difference was observed in the lipid profiles of *C. albicans* biofilms and planktonic cells. Biofilms contained higher levels of phospholipid and sphingolipids than planktonic cells. In the early phase, levels of lipid in most classes were significantly higher in biofilms compared to planktonic cells. The unsaturation index of phospholipids decreased with time, with this effect being particularly strong for biofilms. Inhibition of the biosynthetic pathway for sphingolipid (M(IP)2C) by myriocin or aureobasidin A, and disruption of the gene encoding inositolphosphotransferase 1 (*IPT1*), abrogated the ability of *C. albicans* to form biofilms, suggesting that lipid rafts might be involved in biofilm formation (Lattif et al., 2011). The differences in lipid profiles between biofilms and planktonic *Candida* cells may have important implications for the biology and antifungal resistance of biofilms (Lattif et al., 2011).

In addition, lipid rafts have been found to be important platforms for the concentration of certain virulence factors at the cell surface of pathogenic fungi, to allow efficient access to enzyme substrate and/or to provide rapid release to the external environment. Thus, rafts from the fungal pathogen *C. neoformans* contain the virulence determinant phospholipase B1 (Plb1p), a GPI-anchored protein, and the antioxidant virulence factor Cu/Zn superoxide dismutase (Sod1p; Siafakas et al., 2006). The enzyme Plb1p contains phospholipase B (PLB), lysophospholipase (LPL), and LPL transacylase (LPTA) activities (Chen et al., 1997a,b), and therefore it might affect raft lipid composition.

## LIPID RAFTS AND ION HOMEOSTASIS IN YEAST

The maintenance of ion homeostasis in response to changes in the environment is vital to all living cells. In yeast cells, the active transport of inorganic ions and nutrients relies on the existence of an electrochemical gradient of protons across the plasma membrane. In *S. cerevisiae*, this electrochemical gradient is mainly generated by the essential H^+^-ATPase gene, *PMA1*, which encodes one of the most abundant proteins in the yeast plasma membrane (Serrano et al., 1986). This Pma1p-mediated electrochemical gradient is balanced by the activity of a number of symporters and antiporters, but the high-affinity potassium uptake through the plasma membrane transporters Trk1p and Trk2p, the former being the most biologically relevant potassium transporter, is the major consumer of the gradient (Gaber et al., 1988; Ko and Gaber, 1991; Madrid et al., 1998). Potassium transport into yeast cells results in plasma membrane depolarization, leading to Pma1p stimulation and a concomitant cytosolic alkalinization (Rodriguez-Navarro, 2000). Thus, the regulation of both Pma1p and Trk1p is critical for the modulation of the electrical membrane potential and intracellular pH. Thus, Pma1p and the high-affinity potassium transporters Trk1p and Trk2p are the major determinants of yeast membrane potential and internal pH, and thus should be co-ordinately regulated. The plasma membrane proton ATPase Pma1p is a resident raft protein (Bagnat et al., 2001). The major K^+^ transporters, Trk1p and Trk2p, have also been reported to be present in lipid rafts (Zeng et al., 2004; Yenush et al., 2005). Intracellular pH plays a critical role in modulating the activity of many cellular systems, including those regulating cell death, both in yeasts (Ludovico et al., 2001, 2002; Sokolov et al., 2006) and mammalian cells (Perez-Sala et al., 1995; Gottlieb et al., 1996; Meisenholder et al., 1996).

In *S. cerevisiae*, intracellular pH and K^+^ concentrations affect many cellular activities, including cell growth and death, and thereby they must be tightly controlled through the regulation of the H^+^-pumping ATPase Pma1p and the major K^+^ transporters Trk1p and Trk2p (Yenush et al., 2002 ,^2005^). Pma1p is an electrogenic pump with an optimal pH of 6.5 and therefore is well suited to set the intracellular pH at a neutral value (Portillo, 2000), and together with Trk1p, both systems are the major regulators of cell volume, turgor, membrane potential, and pH in yeast. Potassium accumulation through Trk1p can be harmful to the cell, leading to an increase in turgor pressure and the risk of cell lysis. Trk1p is activated by Hal4p and Hal5p kinases and inhibited by the Ppz1p and Ppz2p phosphatases. Hal3p is a negative regulatory subunit of the Ppz1p Ser/Thr phosphatase (de Nadal et al., 1998), and it has been shown that the inhibition of Ppz1p by Hal3p is pH dependent (Yenush et al., 2005). Interestingly, Ppz1p–Hal3p interaction would act as an intracellular pH sensor, and a model has been proposed for the modulation of H^+^ and K^+^ homeostasis through the regulation of Trk1p activity by intracellular pH (Yenush et al., 2005). At a relatively alkaline pH the interaction between Hal3p and Ppz1p would be destabilized, and hence the Ppz1p phosphatase would act on Trk1p to decrease the potassium uptake into the cell. Thus, following accumulation of K^+^, cells use then a concomitant increase in intracellular pH through the extrusion of protons by Pma1p to downregulate potassium transport.

Ppz1p has been mostly located at the plasma membrane, although some of this protein was also present in internal non-vacuolar membranes, likely the endoplasmic reticulum and/or the nuclear membrane (Yenush et al., 2005). However, unlike Trk1p, Ppz1p was not found in DRMs (Yenush et al., 2005). Nevertheless, because the raft-located Trk1p has been shown to interact with Ppz1p (Yenush et al., 2005), it cannot be ruled out that lipid rafts might transiently be a place where these proteins could interact each other, as shown for other non-raft proteins in mammalian cells that are translocated and recruited in lipid rafts to regulate cell death signaling (Gajate et al., 2004; Gajate and Mollinedo, 2007; Nieto-Miguel et al., 2008; Reis-Sobreiro et al., 2009).

The above data on yeast are of major importance for mammalian cells, as regulation of potassium transport and intracellular pH homeostasis is implicated in many diseases (Shieh et al., 2000), including cancer, and it is plausible to envisage that similar transduction pathways connecting pH and potassium homeostasis might play a critical role in human disease and constitute interesting therapeutic opportunities.

In addition, the plasma membrane Na^+^/H^+^ antiporter Nha1p from the budding yeast *S. cerevisiae*, which plays an important role in intracellular Na^+^ as well as pH homeostasis, by mediating the exchange of Na^+^ for H^+^ across the plasma membrane, has also been shown to associate with lipid rafts (Mitsui et al., 2009). In *lcb1-100* mutant cells, which are temperature-sensitive for sphingolipid synthesis, newly synthesized Nha1p failed to localize to the plasma membrane at the non-permissive temperature, but the addition of phytosphingosine or the inhibition of endocytosis in *lcb1-100* cells restored the targeting of Nha1p to the plasma membrane (Mitsui et al., 2009).

**FIGURE 1:**
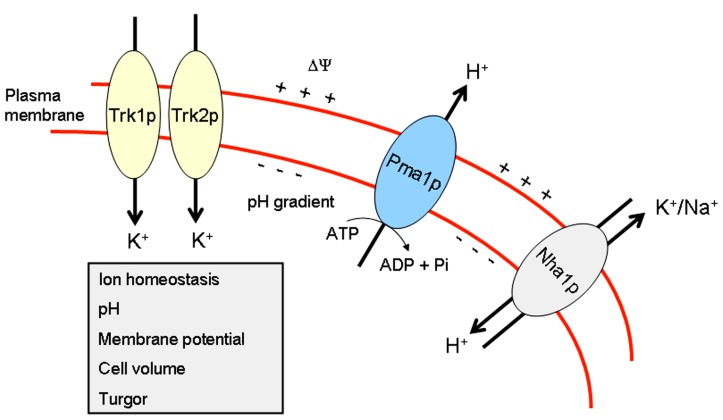
**Ion homeostasis in yeast.** This scheme portrays the major proteins, Pma1p, Trk1p, Trk2p, and Nha1p, involved in maintaining ion homeostasis in *S. cerevisiae*.

Thus, Pma1p, Trk1p, and Nha1p, regulating membrane potential and intracellular pH, are located in lipid rafts in *S. cerevisiae* (**Figure 1**), and they are critical in the modulation of ion homeostasis, by keeping the major monovalent cations (H^+^, K^+^, and Na^+^), mainly through proteins that extrude H^+^ and Na^+^ and import K^+^ ions (**Figure 1**), at the appropriate narrow range of ion concentrations for the proper function of distinct biological processes.

## NUTRIENT TRANSPORTERS IN YEAST LIPID RAFTS

**Table 1** shows a number of proteins that have been located in lipid rafts in *S. cerevisiae*, including proteins involved in ion homeostasis, nutrient transport, mating, stress response, and actin cytoskeleton organization.

**Table 1:** Proteins associated with lipid rafts in *S. cerevisiae*.

Gene	Description	Biological process	Major localization	Technical approach	Reference
*PMA1*	Plasma membrane ATPase	Proton transport, pH regulation	Plasma membrane	DRMGC	Bagnat et al. (2001)
*TRK1*	Potassium transporter	Cellular potassium ion homeostasis	Plasma membrane	DRMGC	Yenush et al. (2005)
*TRK2*	Potassium transporter	Cellular potassium ion homeostasis	Plasma membrane	RTE	Zeng et al. (2004)
*NHA1*	Na^+^/H^+^ antiporter	Ion homeostasis	Plasma membrane	DRMGC	Mitsui et al. (2009)
*TAT2*	Tryptophan transporter	Tryptophan transport	Plasma membrane	DRMGC	Umebayashi and Nakano (2003)
*CAN1*	Arginine permease	Arginine transport	Plasma membrane	DRMGC	Malinska et al. (2003)
*GAP1*	General amino acid permease	Amino acid transport	Plasma membrane	DRMGC	Lauwers and Andre (2006)
*HXT1*	Low-affinity glucose transporter	Glucose transport, hexose transport	Plasma membrane	DRMGC	Lauwers and Andre (2006)
*FUR4*	Uracil permease	Uracil transport	Plasma membrane	DRMGC	Hearn et al. (2003)
*FUS1*	Cell FUSion	Mating	Mating projection tip	DRMGC	Bagnat and Simons (2002)
*FUS2*	Cell FUSion	Mating	Mating projection tip	DRMGC	Bagnat and Simons (2002)
*FIG1*	Factor-induced gene	Mating	Mating projection tip	DRMGC	Bagnat and Simons (2002)
*SHO1*	Transmembrane osmosensor	Osmosensor activity, mating	Plasma membrane, mating projection tip	DRMGC	Bagnat and Simons (2002)
*STE6*	Plasma membrane ATP-binding cassette (ABC) transporter	Peptide pheromone export, mating	Plasma membrane, mating projection tip	DRMGC	Bagnat and Simons (2002)
*PRM1*	Pheromone-regulated membrane protein	Response to pheromone, mating	Mating projection tip	DRMGC	Bagnat and Simons (2002)
*SLG1/WSC1*	Sensor-transducer of the stress-activated PKC1-MPK1	Response to heat and osmotic stress, actin depolarization	Plasma membrane, mating projecting tip	RTE	Lodder et al. (1999)
*RVS161*	Amphiphysin-like protein	Actin cytoskeleton organization, response to osmotic stress and starvation	Actin cortical patch, mating projecting tip	RTE	Balguerie et al. (2002)
*RVS167*	Amphiphysin-like protein	Lipid tube assembly	Actin cortical patch, mating projecting tip	RTE	Germann et al. (2005)
*GAS1*	Glycophospholipid-anchored surface protein/β-1,3-glucanosyltransferase	Fungal-type cell wall organization	Plasma membrane, cellular bud scar	DRMGC	Bagnat et al. (2000)
*HSP30*	Heat shock protein	Stress response, negative regulation of Pma1p	Plasma membrane	DRMGC	Bagnat et al. (2000)
*MRH1*	Membrane protein related to Hsp30p	Unknown	Plasma membrane, mitochondrion	DRMGC	Bagnat et al. (2000)
*NCE2/NCE102*	Non-classical export	Plasma membrane organization	Plasma membrane	DRMGC	Bagnat et al. (2000)

*DRMGC, non-ionic detergent-resistant membrane (DRM) fractions at 4°C followed by flotation in density gradient centrifugation; RTE, resistance to Triton X-100 extraction.*

Several nutrient transporters have been located in lipid rafts in *S. cerevisiae* (**Table 1**). The arginine/H^+^ symporter Can1p (arginine permease) has been found to be present in lipid rafts in *S. cerevisiae* (Malinska et al., 2003). Double labeling experiments with Can1p-GFP and Pma1p-RFP-containing yeast cells showed that these proteins were located in two different non-overlapping membrane domains (Malinska et al., 2003), suggesting the presence of distinct rafts in the same yeast cell. The general amino acid permease Gap1p present at the plasma membrane is also associated with DRMs, and in the absence of sphingolipid synthesis Gap1p fails to accumulate at the plasma membrane and is missorted to the vacuole (Lauwers and Andre, 2006). Likewise, the hexose transporter Hxt1p (low-affinity glucose permease) present at the cell surface was also associated with DRMs (Lauwers and Andre, 2006). The plasma membrane protein uracil/H^+^ symporter Fur4p (uracil permease) has also been reported to be associated with lipid rafts in *S. cerevisiae* (Hearn et al., 2003). The amount of this protein in plasma membrane is highly regulated. Under stress conditions, including heat stress and high concentrations of uracil in the culture medium, Fur4p is degraded by a process that includes phosphorylation, ubiquitination, endocytosis, and transport to the vacuole where the protein is eventually hydrolyzed (Galan et al., 1998; Marchal et al., 2002). Because rafts act as platforms for the integration and modulation of signaling pathways and processes, it could be envisaged that the raft location of Fur4p might be critical for its regulation.

The plasma membrane localization of the tryptophan permease Tat2p is regulated by the external tryptophan concentration and is dependent on lipid rafts. In wild-type cells, Tat2p is transported from the Golgi apparatus to the vacuole at high tryptophan level, and to the plasma membrane at low tryptophan level. However, Tat2p is missorted to the vacuole at low tryptophan concentration in the *erg6*Δ deleted mutant (*ERG6* gene encodes *S*-adenosylmethionine Δ24 methyltransferase, acting in the last steps of ergosterol biosynthesis by converting zymosterol to fecosterol; Umebayashi and Nakano, 2003), and following yeast treatment with the ergosterol biosynthesis inhibitor zaragozic acid, that inhibits squalene synthetase, which catalyzes the first committed step in the formation of cholesterol/ergosterol (Daicho et al., 2007). Likewise, additional proteins, such as Fus1p, a plasma membrane protein required for yeast mating, is excluded from rafts and missorted to the vacuole in the *erg6*Δ mutant (Bagnat and Simons, 2002). These evidences support the view that several plasma membrane proteins can be missorted in *erg6*Δ mutants due to impaired raft association, and this might underlie the mating deficiency (Gaber et al., 1989) and drug hypersensitivity (Kaur and Bachhawat, 1999; Gupta et al., 2003; Maresova et al., 2009) of *erg6*Δ mutants.

Furthermore, as shown in **Table 1**, mating projection-localized proteins Fus2p, Fig1p, Sho1p, Ste6p, and Prm1p have been found to be associated with DRMs (Bagnat and Simons, 2002), supporting a critical role for lipid rafts in the mating process.

## RAFTS AND CELL DEATH IN YEAST

**FIGURE 2:**
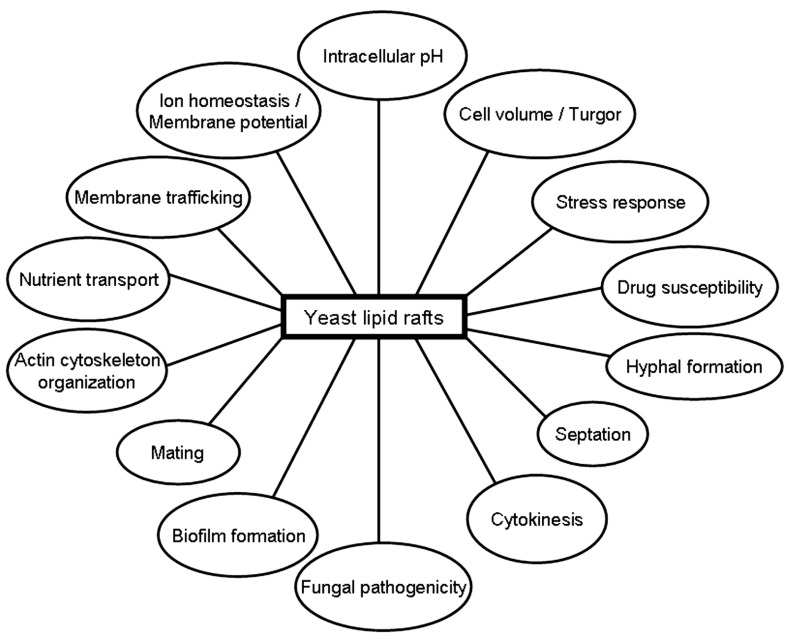
**Putative involvement of lipid rafts in different yeast functions**.

Dysregulation of ion homeostasis mediates cell death, and this represents the mechanistic basis by which a growing number of amphipathic but structurally unrelated compounds elicit antifungal activity (Zhang et al., 2012). Pma1p is displaced from lipid rafts and delivered and degraded to the vacuole upon *S. cerevisiae* incubation with edelfosine (Zaremberg et al., 2005), an amphipathic antitumor ether phospholipid that affects and reorganizes lipid rafts (Gajate et al., 2004, 2009a; Ausili et al., 2008). Because Pma1p is mainly involved in maintaining ion homeostasis and membrane potential in yeast, its displacement from lipid rafts, following treatment of *S. cerevisiae* with the ether phospholipid edelfosine, has been shown to lead to yeast cell death (Zaremberg et al., 2005). Edelfosine has been reported to have affinity for cholesterol, and for cholesterol-rich membranes such as rafts (Ausili et al., 2008; Busto et al., 2008), because of the complementarity of the molecular geometrics of sterols and edelfosine (Busto et al., 2008). Edelfosine induces apoptotic cell death in a wide number of human cancer cells (Mollinedo et al., 1997, 2004, 2010a,b; Gajate and Mollinedo, 2002, 2007; Gajate et al., 2012) through raft reorganization and redistribution of the raft protein content (Gajate and Mollinedo, 2001, 2007; Gajate et al., 2004, 2009a). In human hematopoietic cancer cells, edelfosine treatment leads to the recruitment of apoptotic molecules into raft platforms, thus leading to the emerging concept of an apoptotic “liquid-ordered” plasma membrane platform named as “cluster of apoptotic signaling molecule-enriched rafts” (CASMERs; Gajate and Mollinedo, 2005; Gajate et al., 2009b; Mollinedo and Gajate, 2010a,b). These CASMERs may reduce the apoptotic signal threshold by facilitating and stabilizing protein–protein interactions and cross-talk between signaling pathways, and thereby act as a membrane signaling platforms to launch and catalyze the transmission of apoptotic signals (Mollinedo and Gajate, 2010a,b). The protein composition of these CASMERs includes death receptors and downstream signaling molecules (Gajate and Mollinedo, 2005; Gajate et al., 2009b; Mollinedo and Gajate, 2010a,b). On these grounds, lipid rafts play a major role in the generation of apoptotic signals in mammalian cells. In contrast to mammalian cells, yeasts lack death receptors and most of the typical apoptotic molecules, so the involvement of rafts in the cell death process might be less obvious in yeast. However, lipid rafts seem to be critical structures and scaffolds for the proper function of proteins whose activities are required for the normal function of a yeast cell, including yeast survival and growth (**Figure 2**), such as proteins involved in ion homeostasis and nutrient transport (**Table 1**). In this regard, it might be envisaged that redistribution and displacement of raft-located proteins to non-raft domains might lead to a failure in yeast functioning and eventually to cell death.

In mammalian cells, lipid rafts usually house proteins involved in survival signaling and growth, and thereby their presence is expected to play a role in the proliferation and survival of cancer cells. However, as indicated above, recent evidence in the last few years has also shown the presence of rafts enriched in death receptors and apoptotic molecules, leading to the emerging concept of the proapoptotic CASMER. Thus, at least two major different raft domains leading to survival and cell death can apparently be formed in mammalian cells. However, because yeast cells lack death receptors and the classical mammalian-like caspases, yeast rafts are supposed to be involved only in rather positive outcomes that keep the yeast cell alive. According to this notion, it might be envisaged that lipid raft disruption could facilitate and prompt yeast cell death.

The cationic amphipathic and antiarrhythmic drug amiodarone interacts with lipid membranes to exert their biological effect. In *S. cerevisiae*, toxic levels of amiodarone trigger a transient membrane hyperpolarization, likely through its ability to intercalate into the lipid bilayer (Herbette et al., 1988) altering lipid fluidity (Rosa et al., 2000), which is followed by depolarization, coincident with influx of Ca^2^^+^ and H^+^ that can overwhelm cellular homeostasis and lead to cell death (Maresova et al., 2009). Amiodarone has been shown to have potent fungicidal activity against not only *S. cerevisiae*, but also for species of *Cryptococcus*, *Candida*, *Fusarium*, and *Aspergillus* (Courchesne, 2002). Using a genome-wide screen in a *S. cerevisiae* single-gene deletion library, 36 yeast strains with amiodarone hypersensitivity were identified, including mutants in transporters (*PMR1*, *PDR5*, vacuolar H^+^-ATPase), ergosterol biosynthesis (*ERG3*, *ERG6*, *ERG24*), intracellular trafficking (*VPS45*, *RCY1*), and signaling (*YPK1*, *PTC1*; Gupta et al., 2003). The fact that azole resistant mutants in the ergosterol biosynthesis pathway of *S. cerevisiae* (*erg3*Δ, *erg6*Δ, and *erg24*Δ) exhibited hypersensitivity to amiodarone, suggests that the drug may be particularly effective for treatment against azole-resistant fungal strains (Gupta et al., 2003), which might be defective in raft-mediated processes. In addition, low doses of amiodarone and an azole (miconazole, fluconazole) are strongly synergistic and show potent fungicidal effects in combination (Gupta et al., 2003). These data suggest that lipid raft disruption might favor amiodarone cytotoxic action against yeast.

The initial hyperpolarization seems to be critical for the amiodarone cytotoxic effect. Glucose increases membrane potential by increasing H^+^ pumping activity of the plasma membrane ATPase Pma1p (Serrano, 1983). Downregulation of the H^+^ pump activity of the yeast plasma membrane upon glucose removal (Serrano, 1983) was accompanied by an attenuation of amiodarone-induced Ca^2^^+^ burst, thus protecting against drug toxicity (Muend and Rao, 2008). Amiodarone-induced hyperpolarization was lower in the mutant strain *pma1-105* that has 65% reduction in activity (Perlin et al., 1989). Furthermore, a decrease in membrane potential by glucose removal, addition of salts or in *tok1*Δ, *ena1-4*Δ, or *nha1*Δ mutants, involved in ion homeostasis, protected against amiodarone toxicity, suggesting that initial hyperpolarization was important in the mechanism of antifungal activity (Maresova et al., 2009). Thus, there is a link between membrane hyperpolarization and amiodarone toxicity. Because ion homeostasis and membrane potential is mainly regulated by raft-located proteins, these data also involve these membrane domains in the cytotoxicity of certain fungal drugs.

Interestingly, a recent work by Tulha et al. (2012) has shown that deletion of *GUP1* in *S. cerevisiae* leads to hypersensitivity to acetic acid treatment, high levels of reactive oxygen species (ROS) and reduced lifespan, leading to yeast cell death, likely through necrosis rather than apoptosis. Gup1p is a membrane-bound *O*-acyltransferase involved in remodeling GPI anchors (Bosson et al., 2006), playing an important role in the assembly/integrity of lipid rafts (Ferreira and Lucas, 2008). Because *gup1*Δ mutant is affected in lipid raft integrity/assembly, lipid metabolism, and GPI-anchor remodeling, the above data (Tulha et al., 2012) suggest a role of lipid rafts in yeast cell death process, and in the type of cell death process that ensues reorganization of plasma membrane domains.

Cytotoxicity of the antitumor ether phospholipid edelfosine against *S. cerevisiae* has been shown to be enhanced in yeast mutants defective for *LCB1*, an essential serine palmitoyltransferase that catalyzes the first step in sphingolipid synthesis, and *ERG3*, a sterol C-5 desaturase involved in the final steps of ergosterol synthesis (Zaremberg et al., 2005). On the other hand, *S. cerevisiae* mutants affected in sphingolipid and ergosterol biosynthesis, namely *ipt1*Δ, *sur1*Δ, *skn1*Δ, and *erg3*Δ deletion mutants, are resistant to the azole antimycotics miconazole, mainly due to the role of lipid rafts in mediating intracellular accumulation of miconazole in yeast cells (Francois et al., 2009). Taken together these data suggest a major role of lipid rafts in the cytotoxicity of drugs in yeast.

In this regard, a number of data support that absence of ergosterol, which is one of the major constituents of membrane rafts, has a direct effect on drug susceptibility and morphogenesis of *C. albicans*. Low doses of amiodarone have been reported to be synergistic with fluconazole in fluconazole-resistant *C. albicans* (Gamarra et al., 2010). Ergosterol deficiency in *erg1*Δ mutants led to defects in growth and increased susceptibilities to drugs, including fluconazole, ketoconazole, cycloheximide, nystatin, amphotericin B, and terbinafine in *C. albicans* (Pasrija et al., 2005a,b). Reduced drug efflux activity of the *erg1*Δ mutant was associated with poor surface localization of Cdr1p, suggesting that enhanced passive diffusion and reduced efflux mediated by the ABC transporter Cdr1p increases drug susceptibility. Additionally, conditional *erg1*Δ mutant strains were unable to form hyphae in various media in *C. albicans* (Pasrija et al., 2005a,b). Likewise, the loss of (M(IP)2C) in the *C. albicans* *ipt1*Δ mutant, a sphingolipid biosynthetic gene, resulted in increased sensitivity to drugs like 4-nitroquinoline oxide, terbinafine, *o*-phenanthroline, fluconazole, itraconazole, and ketoconazole. The increase in drug susceptibilities of *ipt1*Δ mutant cells was linked to an altered sphingolipid composition, which appeared to be due to the impaired functionality of Cdr1p, a major drug efflux pump of *C. albicans* that belongs to the ABC superfamily (Prasad et al., 2005). Taken together, the above data indicate that an altered composition of sphingolipid or ergosterol, the major constituents of membrane rafts, affects drug susceptibility and morphogenesis in *C. albicans*.

## ACTIN CYTOSKELETON, RAFTS, AND STRESS RESPONSE

Wild-type *S. cerevisiae* cells depolarize actin following salt stress and repolarize after a period of adaptation (Chowdhury et al., 1992; Balguerie et al., 2002). Two proteins are mainly involved in this process, namely Wsc1p for actin depolarization and the amphiphysin-like protein Rvs161p for actin repolarization (Balguerie et al., 2002). Thus, *rvs161*Δ mutant was able to depolarize actin in response to NaCl stress, but was unable to repolarize afterward, whereas *wsc1*Δ mutants was impaired in depolarizing actin (Balguerie et al., 2002). *RVS161/END6* gene, the budding yeast homolog of amphiphysin (Sivadon et al., 1995; Youn et al., 2010), is associated, in part, with lipid rafts (Balguerie et al., 2002), and co-localizes with actin patches (Balguerie et al., 1999), thus suggesting a link between rafts and actin cytoskeleton in *S. cerevisiae*. Rvs161p is suggested to locate in rafts through a putative association with a raft-bound protein, as Rvs161p has no GPI signal anchor or transmembrane domain, and therefore it cannot be directly integrated in rafts. The *SLG1/WSC1* gene product has also been reported to be partially present in DRMs (Lodder et al., 1999). Clustering of Slg1p/Wsc1p is enhanced under stress conditions, either heat or hypo-osmotic shock, as assessed by single-molecule atomic force microscopy, suggesting its relevance in stress response (Heinisch et al., 2010). Thus, lipid rafts could function as platforms for actin depolarization and actin repolarization in response to stress in *S. cerevisiae*.

In yeast, nutrient starvation leads to entry into stationary phase. *RVS161* (RVS for Reduced Viability upon Starvation) was identified as a critical gene to respond properly to carbon, nitrogen, and sulfur starvation conditions in *S. cerevisiae*, and it has been implicated in the control of cellular viability. Thus, *rvs161*Δ mutant cells die during the stationary phase and show sensitivity to high salt concentrations (Crouzet et al., 1991). *Rvs161*Δ displays a phenotype similar to that shown for the actin mutants: actin cytoskeleton disorganization, random budding of the diploids, loss of polarity, and sensitivity to salt. In addition, *rvs161*Δ, together with mutations in the actin gene, *ACT1*, leads to synthetic lethality (Breton and Aigle, 1998), thus suggesting that actin and Rvs161p are linked in a common functional pathway that is critical for yeast viability under stress conditions. These data might suggest a role of lipid rafts as platforms for the interaction of proteins that are required for yeast survival.

Rvs167p, another amphiphysin-like protein that interacts with Rvs161p to regulate actin cytoskeleton, endocytosis, and viability following starvation and osmotic stress, has been reported to localize to Rvs161p-containing lipid rafts (Germann et al., 2005). Several protein networks involved in diverse cellular functions, including endocytosis/vesicle traffic, converge on Rvs161p-Rvs167p complex (Bon et al., 2000), and thereby Rvs167p-Rvs161p complex might act as a docking platform for proteins involved in the regulation of different biological processes requiring actin cytoskeleton (Germann et al., 2005). In addition, the *rvs* mutants accumulate late secretory vesicles at sites of membrane and cell wall construction, and are synthetic-lethal with the *slt2/mpk1*Δ mutation, which affects the MAP kinase cascade controlled by Pkc1p and is required for cell integrity (Breton et al., 2001). These data support the idea that the RVS proteins, and thereby lipid rafts, are involved in the late targeting of vesicles whose cargoes are required for cell wall construction.

Actin-linking proteins ezrin, moesin, RhoA, and RhoGDI were shown to be recruited into clusters of Fas/CD95-enriched rafts in human leukemic cells upon treatment with the anticancer drug aplidin (Gajate and Mollinedo, 2005). Disruption of lipid rafts and interference with actin cytoskeleton prevented Fas/CD95 clustering and apoptosis, suggesting a major role of actin cytoskeleton in the formation of Fas/CD95 clusters and in the aggregation of proteins in lipid raft clusters in human cancer cells, thus regulating raft-associated signaling events (Gajate and Mollinedo, 2005). There is increasing of evidence of structuring of rafts by the cortical actin cytoskeleton, including evidence that the actin cytoskeleton associates with rafts, and that many of the structural and functional properties of rafts require an intact actin cytoskeleton (Chichili and Rodgers, 2009; Ayling et al., 2012).

In yeast, stabilization of actin by addition of jasplakinolide, by point mutations in the *ACT1* gene, or by deletion of certain genes that regulate F-actin, leads to cell death. Yeast mutant lacking the gene *END3* shows stabilized actin and elevated levels of ROS, this phenotype being dependent on downstream elements of the Ras/cAMP pathway (Gourlay and Ayscough, 2006). Following yeast treatment with methyl-β-cyclodextrin, which depletes sterols from plasma membrane and disrupts lipid rafts, and manumycin A, that blocks prenylation, Ras2 membrane association and the level of ROS were reduced, and cell death progression was inhibited (Du and Ayscough, 2009). These data suggest that lipid rafts in yeast could be somehow related to providing platforms for the generation of stable complexes that could launch pro-cell death signals. This could open the possibility that lipid rafts in yeast could provide appropriate membrane domains for pro-cell death signaling molecules, as it has been recently described in mammalian cells, and not only for survival and growth signaling. However, this pro-cell death view of rafts in yeast remains to be elucidated, and further studies as well as the molecular characterization of the putative processes involved will be required.

## LIPID RAFTS AND EISOSOMES

**FIGURE 3:**
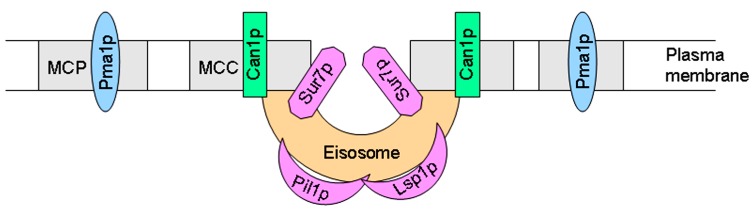
**Schematic view of yeast MCP, MCC, and eisosome membrane subdomains**.

Studies on the budding yeast *S. cerevisiae* have revealed that fungal plasma membranes are organized into different subdomains. Pma1p (plasma membrane H^+^-ATPase) and Can1p (H^+^-arginine symporter) have been located in lipid raft membrane domains, but, as stated above, these proteins occupy two different non-overlapping membrane microdomains (Malinska et al., 2003). Thus, at least two different types of rafts can be distinguished in the yeast plasma membrane. Similarly to what was observed with Can1p, a family of integral membrane proteins, including Sur7p, Ynl194p, and Ydl222p, were visualized in cortical patches in *S. cerevisiae* (Young et al., 2002). Current evidence suggests the existence of at least two subcellular compartments in the yeast plasma membrane, namely a raft-based membrane compartment represented by a network-like structure housing Pma1p, and another raft-based membrane compartment that houses a number of proton symporters (Can1p, Fur4p, Tat2p). These two raft domains, also named as the membrane compartment occupied by Pma1p (MCP) and membrane compartment occupied by Can1p (MCC; Malinska et al., 2004; Grossmann et al., 2007), apparently require different lipids to keep their respective protein compositions (**Figure 3**). The proper sorting of Pma1p has been reported to be more dependent on sphingolipids, ceramide, and the C26 acyl chain that forms part of the ceramide (Lee et al., 2002; Gaigg et al., 2005, 2006). However, sterols are required for the correct targeting of Tat2p (Umebayashi and Nakano, 2003). Thus, these data suggest the putative existence of raft domains more enriched in either sphingolipids or sterol-rich in the yeast membrane, each one containing a specific set of proteins, and this compartmentalization or lateral segregation seems to be dependent on the membrane potential (Grossmann et al., 2007). Plasma membrane depolarization caused reversible dispersion of the H^+^ symporters previously present in 300-nm patches (Grossmann et al., 2007). In addition, yeast plasma membrane seems to contain an additional subdomain named eisosomes (from the Greek “*eis*,” meaning into or portal, and “*soma*,” meaning body), which are immobile protein complexes, composed mainly of the cytoplasmic proteins Pil1p and Lsp1p at the plasma membrane that mark static sites of endocytosis (Walther et al., 2006). Pil1p and Lsp1p form punctuate clusters (eisosomes) on the cytoplasmic surface of the plasma membrane at MCC sites (Walther et al., 2006) and associate with the plasma membrane via their BAR domains (named for the Bin/Amphiphysin/Rvs proteins), that bind membranes and promote curvature (Zimmerberg and McLaughlin, 2004). Eisosomes form at the sites of invaginations in the plasma membrane, being flanked by Can1p-rich MCC domains at the upper edges of the furrows, whereas Pil1p is located at the bottom of the furrow (Stradalova et al., 2009; Douglas et al., 2011) and Sur7p, a protein involved in endocytosis, seems to be at the boundary between MCC and eisosomes (**Figure 3**). Microscopic and genetic analyses link these stable, ultrastructural assemblies, named eisosomes, to the endocytosis of both lipid and protein cargoes in cells, and are mainly composed of BAR domain proteins (Douglas et al., 2011; Olivera-Couto et al., 2011). Eisosomes have been suggested to function as organizing sites for endocytosis (Toret and Drubin, 2006). Thus, degradation of the arginine permease Can1p induced by excess of its substrate required first Can1p release from MCC patches, and only then it was endocytosed (Grossmann et al., 2008), thus suggesting that the protein is to a large extent unavailable for endocytosis and subsequent degradation as long as it stays in the protective area of MCC (Malinsky et al., 2010). In this regard, rapidly moving endocytic marker proteins avoid raft domains, and consequently the raft domain-accumulated proton symporters show a reduced state of substrate-induced endocytosis and turnover (Malinsky et al., 2010). Genetic analysis of the MCC/eisosome components indicates that these domains broadly affect overall plasma membrane organization (Douglas et al., 2011). The analysis of the major constituents of eisosomes, i.e., BAR proteins, is of major importance also in mammalian cells. BAR family proteins contribute to a range of cellular functions characterized by membrane and cytoskeletal remodeling, inducing membrane curvature and recruitment of effector proteins, with important consequences in several human disorders, including cancer cell invasiveness, as well as immune and neurologic disorders (Chen et al., 2012).

## PERSPECTIVES

Recognition of the presence of distinct domains at the cell membrane has been one of the most significant scientific achievements in the last decades. Lipid raft membrane domains are gaining momentum in current biology, and they seem to regulate a wide number of critical processes. Lipid rafts are variable in size and composition, and can change in a highly dynamic way both by recruiting and expelling components as well as by coalescing smaller raft units, forming large clusters. The presence of these membrane domains in all eukaryotic cells opens new ways to study the physiological role of rafts in distinct biological systems. The existence of lipid rafts in yeast has provided an excellent way to study the role of these membrane domains in different biological processes, due to the remarkable yeast genetic tractability. Thus, changes in ergosterol and sphingolipid composition, the major raft constituents in yeast, by disrupting key metabolic genes lead to improper surface localization of major proteins involved in keeping yeast viable against some external aggression, including proteins involved in ion homeostasis, drug efflux, and stress response. Yeast lipid rafts house proteins that are critical for the regulation of membrane potential, intracellular pH, and nutrient transport. In fact, the plasma membrane potential is mainly considered as the driving force for ion and nutrient translocation in yeast, and the correct location of the proteins involved in these processes in lipid rafts is crucial for the proper functioning of the yeast. Lipid rafts could also behave as scaffolds where proteins dock and concentrate to either launch signals to be transmitted to other parts of the cell or to build new structures required for cell integrity or cell division. Thus, yeast cells constitute an excellent biological system to analyze the role of lipid rafts in both survival and cell death responses, in spite of lacking most apoptotic molecules present in mammalian cells. Key proteins for yeast survival and proper yeast functioning are localized in lipid rafts, and their displacement from raft domains leads to cell death. In addition, drugs interacting with sterols affect lipid raft composition and membrane integrity and are detrimental to yeast. In addition, yeast plasma membrane contains at least three distinct subdomains that appear to have specialized functions, and they could interact each other. Understanding the dynamic structure of lipid rafts in yeast, as well as their mobility, composition, and biological role, will be of an inestimable value in getting a better insight into the role of these membrane domains in survival and cell death signaling. This insight will recognize the importance of membrane lipids in cellular function, highlighting lipid rafts as a new and promising therapeutic target (Mollinedo and Gajate, 2006), and could be useful for the search of novel antifungal agents and, following extrapolation to mammalian cells, to hopefully set up the underlying bases for the treatment of human diseases. Membrane compartmentalization in lipid rafts plays a key role in signaling process in mammalian cells, and this aspect might also be true in yeast as a plethora of processes seem to involve proteins located in these yeast membrane domains. Thus, a better knowledge of the yeast lipid raft composition and function might help us to gain insight in the regulation of critical processes regarding cell fate, which might be extrapolated to other organisms and could be valuable to conceive new approaches in the treatment of human diseases where cell death and survival are critical, such as cancer and neurodegenerative diseases.

## Conflict of Interest Statement

The author declares that the research was conducted in the absence of any commercial or financial relationships that could be construed as a potential conflict of interest.
